# Synthesis of Poly(Lactic Acid-*co*-Arginine) and Construction of Its Ternary Phase Diagram for Nonsolvent Induced Phase Separation

**DOI:** 10.3390/ma18204816

**Published:** 2025-10-21

**Authors:** Yinying Zhu, Hongxia Yan, Bei Wang, Zihan Shangguan, Junyan Yao

**Affiliations:** 1 School of Chemistry and Chemical Engineering, Northwestern Polytechnical University, Xi’an 710129, China; 2 Queen Mary University of London Engineering School, Northwestern Polytechnical University, Xi’an 710129, China

**Keywords:** poly(lactic acid-*co*-arginine), hydrophilic, phase separation, ternary phase diagram

## Abstract

L-arginine, a basic amino acid, exhibits high biocompatibility, reactivity, and absorbability. It was selected as the co-polymer modification monomer for L-lactic acid with the objective of enhancing the hydrophilicity of poly(lactic acid) (PLA), neutralizing the acidity of PLA degradation products, and regulating the degradation cycle. The copolymer poly(lactic acid-*co*-arginine) (PLAA) was synthesized by direct melting polycondensation of L-arginine and L-lactic acid, and the structures and properties of PLAA were characterized. The results indicated the presence of –NH_2_, –NH–, and NH= in the molecular chain of the copolymer PLAA. Furthermore, the PLAA was identified as an amorphous copolymer. The “PLAA/CHCl_3_/C_6_H_14_” ternary phase diagram was constituted by nonsolvent-induced phase separation (NIPS) by selecting chloroform (CHCl_3_) as a good solvent and n-hexane (C_6_H_14_) as a nonsolvent. The phase diagram displays three distinguishable regions: the homogeneous zone, the metastable zone, and the phase separation zone. These regions are identified by the binodal and spinodal curves. The ternary phase diagram establishes a theoretical foundation for the preparation and processing of PLAA nanoparticles, composite materials, and porous fibers or membranes.

## 1. Introduction

Poly(lactic acid) (PLA) is extensively utilized in various clinical medicine applications due to its exceptional biocompatibility and biodegradability [1,2,3,4]. However, due to its long degradation cycle and acidic degradation products, PLA may create a localized acidic environment in the body and trigger inflammatory reactions [5,6]. Amino acids, defined as non-toxic and safe bio-friendly substances, have been demonstrated to maintain the biocompatibility of poly(lactic-co-amino acid) copolymers. In addition, they have been shown to enhance the hydrophilicity of these copolymers and adjust their degradation cycle [7]. The acidic by-products resulting from PLA can be partially neutralized if a basic amino acid is selected [8].

The nonsolvent-induced phase separation (NIPS) process requires the selection of suitable good solvents and nonsolvents for the purpose of collectively preparing polymer solutions and constructing ternary phase diagrams. The ternary phase diagram is derived from the visual identification of the phase separation boundaries of polymer solutions [9,10]. This process is of significance for guiding the subsequent production and processing of polymers in the form of fibers and porous membranes, and the resulting porous materials have broad application prospects in biomedicine, food preservation, and other fields [11,12,13,14,15].

In this paper, L-arginine was selected as the copolymerization-modified monomer with lactic acid. Subsequently, the amorphous poly(lactic acid-*co*-arginine) (PLAA) was synthesized, and its chemical structure and molecular weight were characterized. In addition, a visual investigation was conducted into the dissolution and phase separation of different concentrations of PLAA in various ratios of CHCl_3_/C_6_H_14_ mixed solvents. In this investigation, chloroform (CHCl_3_) was chosen as the good solvent, and n-hexane (C_6_H_14_) was selected as the nonsolvent. The ternary phase diagram of “PLAA/CHCl_3_/C_6_H_14_” was then constructed by NIPS. This revealed the phase transition law and interaction mechanism of PLAA in solvent and nonsolvent systems. It also provided a theoretical basis for the design of polymer dissolution, sedimentation, and porous material processing. The research on the PLAA phase diagram is conducive to the design of the formulation and process parameters for the preparation of the porous materials of PLAA and its blends.

## 2. Materials and Methods

### 2.1. Experimental Materials

L-lactic acid (88%) and L-arginine were supplied by Shanghai Maclean’s Biochemical Technology Co., Ltd. (Shanghai, China), chloroform and acetone were purchased from Chengdu Kelong Chemical Co., Ltd. (Chengdu, China), and phosphoric acid (98 wt%), absolute alcohol, and n-hexane were provided by Guangdong Guanghua Science and Technology Co., Ltd. (Shantou, China).

### 2.2. Synthesis of PLAA

The purified L-lactic acid and L-arginine, with a molar ratio of 85:15, were added to a three-necked flask. The reaction was catalyzed by 0.98 wt% phosphoric acid. The solid substrate was not dissolved prior to the reaction. Subsequently, the mixture was stirred and heated to 155 °C for 15 h, with a vacuum and nitrogen atmosphere alternating every 30 min to remove polymerization byproducts and water. Subsequent to the completion of the reaction, the reaction product was cooled and dissolved in chloroform to form the saturated solution. The saturated solution was precipitated by acetone, and the product was purified after three cycles of precipitation. The purified solid was then subjected to vacuum drying at a temperature of 40 °C for a period of 48 h. This process resulted in the formation of a yellow-brown solid product, which was identified as PLAA. The yield of the product was approximately 69%.

### 2.3. Characterization of PLAA

The molecular chain structure and groups of PLAA were analyzed by Fourier transform infrared spectroscopy (FTIR; Thermo Scientific, Waltham, MA, USA), nuclear magnetic resonance (NMR; Bruker BioSpin, Billerica, MA, USA) (CDCl_3_ as solvent, 27 °C), and X-ray photoelectron spectroscopy (XPS; Kratos Analytical, Stretford, UK).

The molecular weight and molecular weight distribution of PLAA were determined by gel permeation chromatography (GPC-PL50; Waters Corporation, Milford, MA, USA) with N,N-Dimethylformamide (DMF) as solvent at 25 °C, a solution concentration of 4 mg mL^−1^, a flow rate of 1.00 mL min^−1^, and a column height of 650 mm.

Glass transition temperature (*T_g_* ) of PLAA was performed by differential scanning calorimetry (DSC; Mettler Toledo, Hong Kong, China). Each sample of about 5 mg was heated from −20 to 200 °C with the heating rate of 10 °C min^−1^ under nitrogen first. After staying for 5 min, the blend was cooled to −20 °C at the cooling rate of 10 °C min^−1^. Subsequently, the sample was heated from −20 °C to 200 °C at the heating rate of 10 °C min^−1^ after staying for 5 min at the end of the cooling period.

The water contact angle θ of PLAA was determined by a contact angle tester (Dataphysics Corporation, Riverside, CA, USA).

### 2.4. Preparation of “PLAA/CHCl_3_/C_6_H_14_” Ternary System

Chloroform and n-hexane were selected as the good solvent and nonsolvent of PLAA, respectively, based on their distinct solubility characteristics. In this experiment, the temperature and pressure were fixed at 25 °C and atmospheric pressure (101.325 kPa). The phase separation process was driven by the concentration of the components, *i.e*., the concentration of PLAA in CHCl_3_ (wt%) and the ratio of CHCl_3_/C_6_H_14_ (*v*/*v*). The ternary systems of PLAA/CHCl_3_ and CHCl_3_/C_6_H_14_ were investigated at varying concentration ratios. The CHCl_3_/C_6_H_14_ (*v*/*v*) ratio was arranged at 0.15 intervals near the boundary to precisely define the liquid-liquid and solid–liquid separation boundaries in the phase diagram. Various concentrations of PLAA/CHCl_3_ solutions were prepared, then stirred and left to stand for 24 h to ensure complete dissolution of PLAA in CHCl_3_. Subsequently, the requisite volume of C_6_H_14_ was slowly added to the solution with vigorous shaking to ensure complete miscibility with CHCl_3_ and C_6_H_14_. Finally, the solutions were sealed and stored at a constant temperature of 25 °C and atmospheric pressure (101.325 kPa) for up to 14 days under the condition of daily visual inspection.

Visually apparent increases in ambiguity are indicative of turbidity or liquid-liquid phase separation, while precipitation is recorded as solid–liquid phase separation. Based on the observed phase separation of the ternary system, the “PLAA/CHCl_3_/C_6_H_14_” ternary phase diagram was drawn using OriginPro 2018C (64-bit) SR1 b9.5.1.195.

## 3. Results and Discussion

### 3.1. Molecular Weight and Polymerization Mechanism of PLAA

The PLAA has the sole relative molecular mass peak detected by GPC. The *M_w_* is 3.94 × 10^4^ g mol^−1^, *M_n_* is 3.36 × 10^4^ g mol^−1^, and the polydispersity index (PDI) is 1.17.

**Figure 1:**
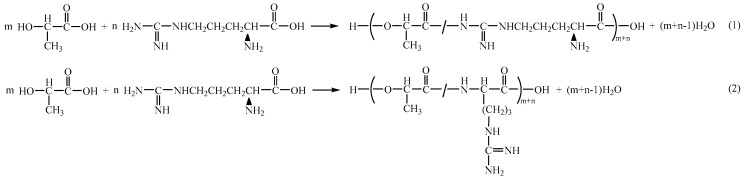
Copolymerization reaction formulae of L-(lactic acid) and L-arginine.

The primary chemical reactions involved in the process of melting copolymerization of L-lactic acid and L-arginine encompass self-polymerization of L-lactic acid, self-polymerization of L-arginine, and condensation polymerization of lactic acid and arginine. The arginine molecule contains two kinds of -NH_2_ (*ω*-amino and *α*-amino), which can undergo condensation polymerization with lactic acid to form two distinct products, as illustrated in Figure 1.

In arginine, the *ω*-amino group exhibits heightened reactivity, preferentially reacting with the carboxyl group to form an amide bond [16,17]. Therefore, the *ω*-amino group is prone to react with the carboxyl group of lactic acid or another arginine molecule by condensation during the polymerization process, so the reaction Formula (1) is more likely to occur.

### 3.2. Chemical Structure Characterization of PLAA

#### 3.2.1. FTIR Characterization of PLAA

**Figure 2:**
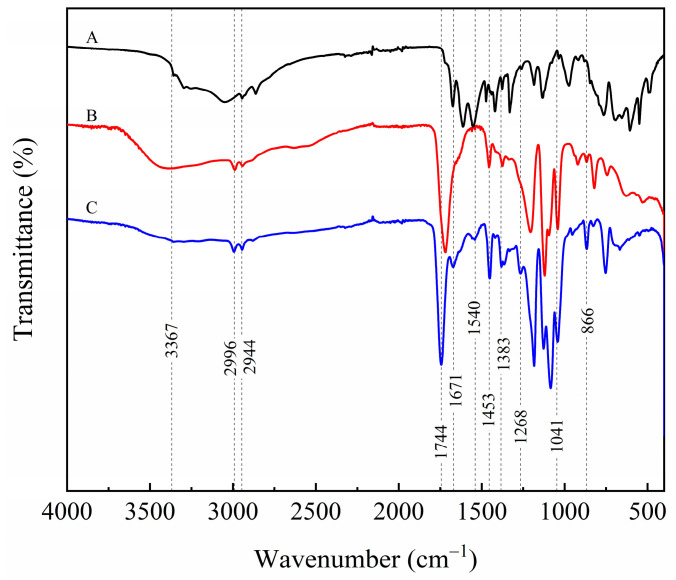
Infrared spectrum of each substance. A: L-arginine; B: L-lactic acid; C: PLAA.

The FTIR spectra of L-lactic acid, L-arginine, and PLAA are shown in Figure 2.

As can be seen from Figure 2, for the copolymer PLAA, there are N-H and O-H stretching vibration peaks near 3367 cm^−1^, an asymmetric stretching vibration peak of –CH– at 2996 cm^−1^, and a peak near 2944 cm^−1^ for the symmetric stretching vibration peak of –CH–. The peak of the stretching vibration of the carbonyl (C=O) bond in PLA chain segments appears near 1744 cm^−1^. In addition, the presence of absorption bands at 1671 cm^−1^ and 1540 cm^−1^ corresponds to the Amide I and Amide II bands, respectively. The in-plane bending vibration peaks of 1453 cm^−1^ and 1383 cm^−1^ represent –CH– and –CH_3_ separately, and the vibrational absorption peak of 1268 cm^−1^ means the amide III band (C–N). The peak near 1041 cm^−1^ is the stretching vibrational peak of the amine C–N bond, and near 866 cm^−1^ is the stretching vibrational absorption of the –C–C– bond. The absorption frequencies of the chemical bonds are the same as those in the relevant literature [18].

The Amide I band (~1671 cm^−1^), which arises from the stretching of the C=O within the newly formed amide bond (–CONH–), and the Amide II band (~1540 cm^−1^), associated with the N–H bending vibration, confirm the successful formation of the amide structure between PLA and L-arginine chain segments. The above analysis can verify that the chemical structure of the obtained product is consistent with that in Figure 1.

#### 3.2.2. The ^1^H-NMR and ^13^C-NMR Spectra of PLAA

**Figure 3:**
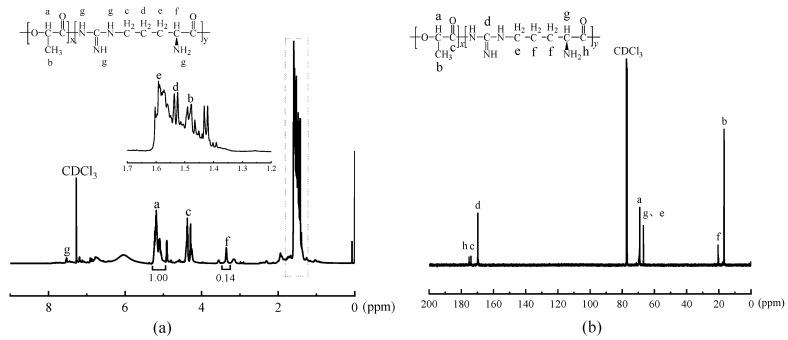
(**a**) ^1^H-NMR spectrum of PLAA, (**b**) ^13^C-NMR spectrum of PLAA.

The NMR spectra of the copolymerization product PLAA were obtained using deuterated chloroform (CDCl_3_) as the solvent. The ^1^H-NMR and ^13^C-NMR spectra were analyzed, and the results were presented in Figure 3.

The ^1^H-NMR spectrum of PLAA is presented in Figure 3a, in which the peak at *δ* 7.47 ppm indicates the H on –NH– and *δ* 5.16 ppm shows the H on –O–CH–CO–. The value of *δ* 4.30 ppm represents the H of -CH_2_- in –NH–CH_2_–, and the peak at *δ* 3.28 ppm represents the H of –CH– in –CH–NH_2_. Additionally, there are peaks at *δ* 1.52 ppm, *δ* 1.46 ppm and *δ* 1.41 ppm, which correspond to the H of –CH_2_– in two different chemical environments on –CH_2_–CH_2_– and the H in –CH_3_, respectively.

Figure 3b shows the ^13^C-NMR spectrum of PLAA, where the chemical shift at *δ* 175.20 ppm denotes the C on -COOH and the peak at *δ* 173.98 ppm evinces the C on –NH–C(=NH)–NH–. The value of *δ* 169.70 ppm indicates the C on –CO–O– and –CO–NH–, the peaks at *δ* 69.12–69.51 ppm represent the C on –CH– in –O–CH–C=O, and the value of *δ* 66.82 ppm denotes the C of –CH_2_– and –NH–CH_2_– in –CH_2_–NH_2_. The chemical shift at *δ* 20.48–20.51 ppm indicates the C on –CH_2_–CH_2_–, and *δ* 16.67–16.89 ppm represents the C on –CH_3_. The above results indicate that the chemical structure of the obtained products is consistent with that of the copolymer of the reaction formula (1) in Figure 1.

The lactic acid units contain methyl (–CH_3_) and methyne (–CH–) groups, whereas the arginine units contain methylene(–CH_2_–) and methyne groups. By calculating the peak areas of the methyne group (peak a) in lactic acid and the methyne group (peak f) in arginine, the proportion of each repeating unit in the PLAA can be obtained. The area ratio of peak a and peak f is 1:0.14, indicating that the ratio of lactic acid unit and arginine unit in the copolymer PLAA is 88:12, while the theoretical ratio of lactic acid unit and arginine unit in the PLAA is 85:15 based on the monomer feed ratio. This indicates that the actual amount of arginine units in PLAA is marginally lower than the theoretical content, suggesting that the reactivity of the two monomers of lactic acid and arginine differs in the copolymerization reaction, with arginine exhibiting slightly less reactivity. The proportion of arginine chain segments in copolymer PLAA is less than the theoretical value because a small amount of arginine did not participate in the copolymerization reaction or was synthesized into low molecular weight self-polymers or copolymers, which were removed during the purification process in cold water.

#### 3.2.3. XPS Analysis of PLAA

**Figure 4:**
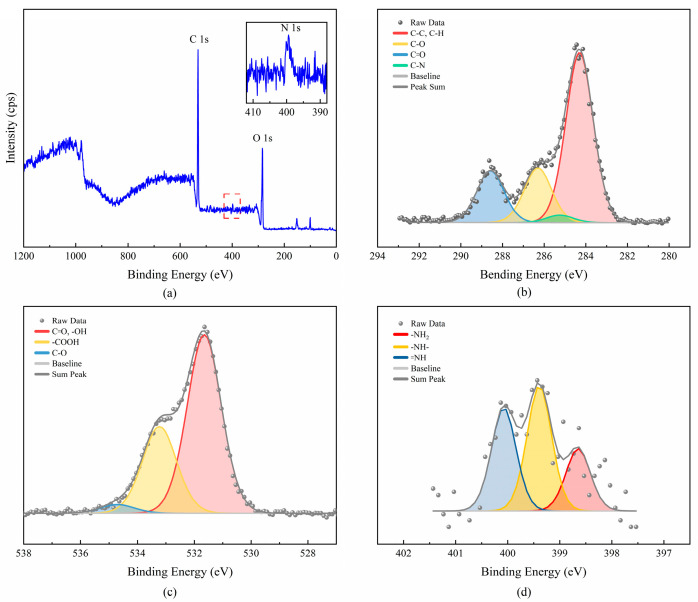
XPS spectrum of PLAA and peak-differentiating spectra of C 1s, O 1s and N 1s: (**a**) wide scan spectrum, (**b**) C 1s peak-differentiating spectra, (**c**) O 1s peak-differentiating spectra, (**d**) N 1s peak-differentiating spectra.

The XPS full spectrum and the atom peak-differentiating spectra of the copolymer PLAA are shown in Figure 4 to further investigate the connection mode of each atom in the molecule.

Figure 4a exhibits characteristic peaks of O 1s, N 1s, and C 1s at 531.23 eV, 398.23 eV, and 284.23 eV, respectively. As shown in Figure 4b, the C 1s spectrum of PLAA can be divided into four peaks, which correspond to the five binding modes of C–H, C–C (at 283.30 eV), C–O (at 286.30 eV), C=O (at 288.58 eV), and C–N (at 285.25 eV) groups in the molecular chains of the copolymer. The peak positions of chemical bonds in the PLAA are consistent with those described in the relevant literature [19,20,21]. The presence of the C–N structure indicates the successful copolymerization of the lactic acid and arginine. Figure 4c displays three differentiating peaks of the element O in PLAA, which correspond to the four binding modes of C=O, –OH (531.65 eV), –COOH (533.23 eV), and C–O (535.24 eV), respectively. As shown in Figure 4d, the distinct nitrogen signal present in the PLAA can be differentiated into three peaks, corresponding to the three binding modes of -NH_2_ and =NH in the arginine molecule, and –NH– in the –CONH– structure in the amide group. The spectrum shows peaks at 398.65 eV, 399.39 eV, and 400.07 eV, which correspond to the –NH_2_, –NH–, and =NH structures, respectively. The presence of the -NH- structure confirms the copolymerization of L-lactic acid and L-arginine.

The FT-IR, ^1^H-NMR, ^13^C-NMR, and XPS spectra of copolymer confirmed the presence of amino, amide, ester, methyl, and methyne groups in the structure of PLAA, which contained the major groups of the lactic acid (ester, methyl and methyne groups) and those of the arginine (amino and amide groups). Therefore, it is inferred that the copolymer PLAA was synthesized by the copolymerization of L-lactic acid and L-arginine. The degradable products of arginine segments in the copolymer PLAA are basic, which can neutralize the acidic environment resulting from lactic acid segment degradation.

#### 3.2.4. Racemization of Lactic Acid Segments in PLAA

**Figure 5:**
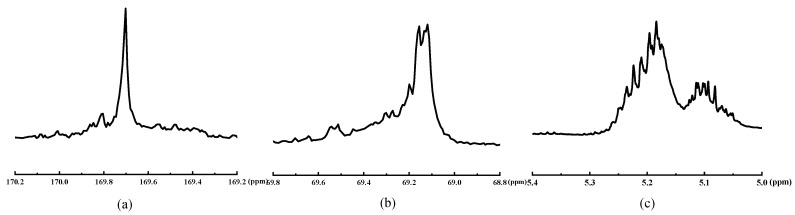
(**a**) carbonyl ^13^C-NMR of lactic acid segments in PLAA; (**b**) methyne ^13^C-NMR of lactic acid segments in PLAA; (**c**) methyne ^1^H-NMR of lactic acid segments in PLAA.

The high-resolution NMR spectra can be used to analyze the stereoregularity and racemization of homopolymers or copolymers of poly(lactic acid), poly(ε-caprolactone), and poly(glycolic acid) [8]. The chemical shift at *δ* 169.5 ppm (carbonyl) and *δ* 69 ppm (methyne) in the ^13^C-NMR spectrum and the value of *δ* 5.16 ppm (methyne) in the ^1^H-NMR spectrum were analyzed to determine the stereoregularity and racemization of the lactic acid segments in PLAA. Based on the principles, the PLAA copolymerization reaction mechanism would be explored. Figure 5 displays the ^13^C-NMR and ^1^H-NMR spectra of lactic acid chain segments in copolymer PLAA.

The results of relevant literature [8,22,23] revealed that the poly(L-lactic acid) (PLLA) molecular chain displayed a semi-crystalline state with a high degree of crystallinity. Both the methyne and carbonyl groups in the ^13^C-NMR spectrum exhibited a sharp singlet, while the methyne group in the ^1^H-NMR spectrum showed a typical quartet. According to Figure 5, the methyne and carbonyl peaks in the lactic acid chain segments of PLAA displayed several peaks of different sizes with obvious splitting and shifting in the ^13^C-NMR spectrum. Similarly, the peaks of the methyne group in the ^1^H-NMR spectrum exhibited evident deformation. The results indicated that the lactic acid chain segments of PLAA exhibited stereo-configurations, demonstrating that ester exchange and chain transfer reactions occurred during the copolymerization process, resulting in obvious racemization. The lactic acid chain segments in PLAA were found to be syndiotactic or atactic. This observation suggests that PLAA is challenging to crystallize and tends to exhibit an amorphous state on a macroscopic scale. This hypothesis could be further substantiated through DSC analysis.

### 3.3. DSC Analysis of PLAA

**Figure 6:**
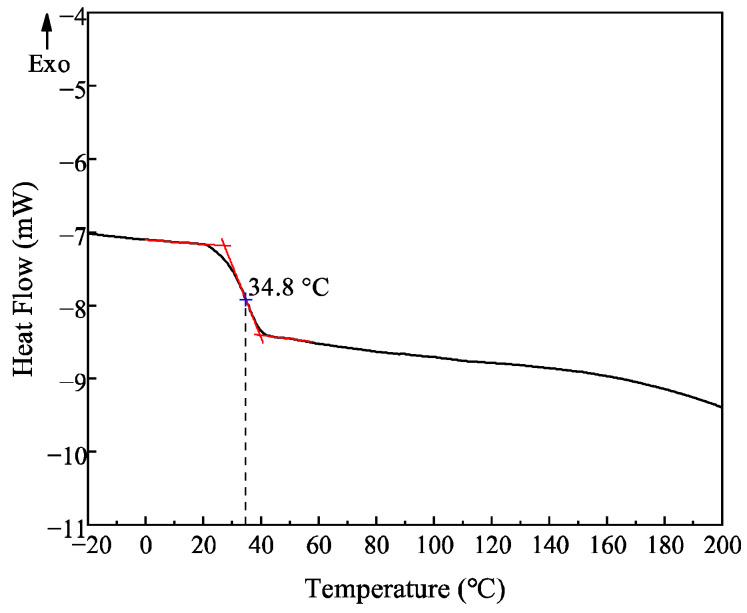
DSC curve of PLAA.

The DSC analysis of PLAA was characterized by differential scanning calorimetry, and the result is shown in Figure 6.

As can be seen from the curve in Figure 6, the copolymer PLAA has a single glass transition temperature of 34.8 °C without obvious crystallization and melting peaks, indicating that PLAA is an amorphous polymer, which is consistent with the racemic analysis results of lactic acid chain segments in PLAA.

### 3.4. Hydrophilic Property Analysis of PLAA

The hydrophilicity of various substances can be effectively estimated by measuring the water contact angle of materials. PLA has a water contact angle of 78°, which is due to its nature as a hydrophobic polymer that lacks hydrophilic groups. Its -CH_3_ side chains also contribute significantly to its hydrophobicity. However, the water contact angle of PLAA is 45°. This is because it is a copolymer of lactic acid and arginine, and its molecular chain contains both hydrophilic (amino and amide) and lipophilic (ester) groups. Therefore, it is a hydrophilic and lipophilic polymer. The addition of arginine enhances the hydrophilicity of PLA.

### 3.5. Construction of “PLAA/CHCl_3_/C_6_H_14_” Ternary Phase Diagram

**Figure 7:**
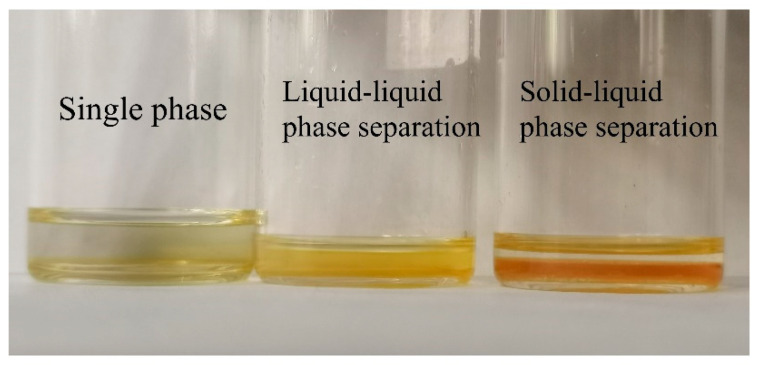
Visual results of PLAA/CHCl_3_/C_6_H_14_ ternary system.

The phase separation behavior and the mechanisms of PLAA in mixed solvents was explored and thereby the “PLAA/CHCl_3_/C_6_H_14_” ternary phase diagram was constructed in this research to guide the solvent-based processing of PLAA composed of chloroform and n-hexane, and realize the regulation of the morphology and size of nanoparticles or porous materials, so as to optimize the performance and application of materials. PLAA is soluble in water, ethanol, chloroform, and N,N-dimethylformamide, but insoluble in n-hexane. Chloroform and n-hexane are miscible. Consequently, the ternary system was established as PLAA/CHCl_3_/C_6_H_14_. The ternary system “PLAA/CHCl_3_/C_6_H_14_” was observed visually at different concentration ratios, as depicted in Figure 7. If the solution remained clear and transparent, it was classified as a single homogeneous phase and recorded as “S” (single phase). If the solution was visibly blurred, it was considered as liquid-liquid phase separation and denoted as “L” (liquid-liquid phase separation). If there was precipitation in the system, it was regarded as solid–liquid phase separation and labeled as “P” (precipitation, solid–liquid phase separation). These observations were shown in Table 1.

Table 1 presents that the potential for phase separation increases with higher concentrations of PLAA in CHCl_3_ (PLAA/CHCl_3_, wt%) and the C_6_H_14_ and CHCl_3_ volume ratio (C_6_H_14_/CHCl_3_, *v*/*v*) in the ternary system. For example, liquid-liquid phase separation occurs for the solution (15.25 wt%, 0.5 *v*/*v*), so it can be denoted that the phase separation would occur for the solutions with higher concentrations of 15.25 wt% PLAA in CHCl_3_ and higher volume ratios of 0.5 *v*/*v* C_6_H_14_/CHCl_3_.

In the ternary phase diagram, the mass concentrations of PLAA, CHCl_3_ and C_6_H_14_ in the ternary system are calculated in Equation (1), Equation (2) and Equation (3), respectively.

(1) PLAA% wt%=m1m1+m2+m3×100=100(XPLAA)100+100·(ρ3·VH/C·V2)(m1+m2)=100(XPLAA)100+100·(ρ3·VH/C·m2)ρ2(m1+m2)=100(XPLAA)100+(0.6601.471)·VH/C·(100−XPLAA)=222.88(XPLAA)222.88+VH/C(100−XPLAA)

(2) $${CHCl}_{3}\%\;\left(wt\%\right)=\frac{m_{2}}{m_{1}+m_{2}+m_{3}}\times 100=\frac{222.8(100-X_{PLAA})}{222.8+V_{H/C}(100-X_{PLAA})}$$

(3) $$C_{6}H_{14}\%\;(wt\%)=100-PLAA\%-{CHCl}_{3}\%$$

In the equations, $X_{PLAA}$ is the mass concentration of PLAA in CHCl_3_ (%); $V_{H/C}$ is volume ratio of C_6_H_14_/CHCl_3_; *m_i_*, *ρ_i_*, and *V_i_* are mass, density, volume of the *i* substance, where 1, 2 and 3 stand for PLAA, CHCl_3_ and C_6_H_14_, respectively. Wherein, the density of the PLAA is 0.984 g cm^−3^.

**Figure 8:**
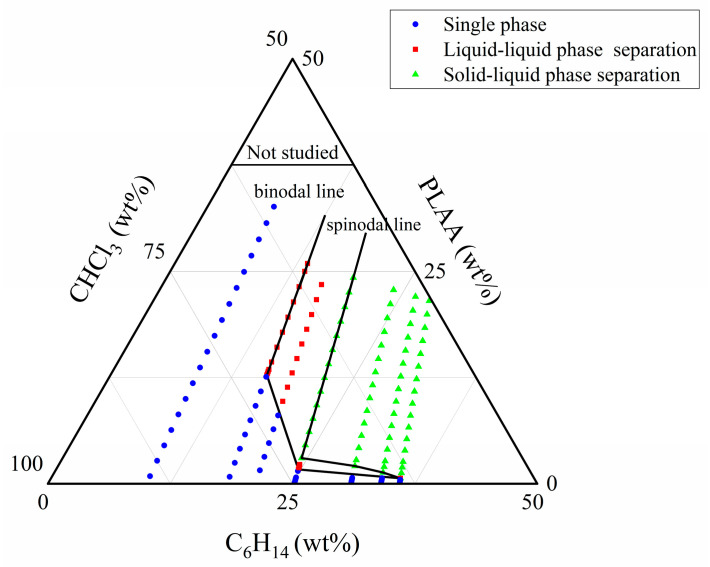
“PLAA/CHCl_3_/C_6_H_14_” ternary phase diagram.

According to the above equations, all composition points in ternary systems can be obtained and plotted on a ternary phase diagram. The binodal and spinodal lines are sketched by connecting the boundary points. The “PLAA/CHCl_3_/C_6_H_14_” ternary phase diagram is constructed, and the results are shown in Figure 8.

In Figure 8, the boundary between the single-phase and liquid-liquid phase separation zones is a binodal line, and the boundary between the single-phase or liquid-liquid phase separation zone and the solid–liquid phase separation zone is a spinodal line. The constituent regions proximate to the binodal line boundary are predicted to manifest metastable behavior, with the true binodal line potentially situated marginally below the boundary delineated by experimental observations. This suggests that the single-phase zone is more extensive, and the binodal line is positioned at a higher PLAA wt% and a lower C_6_H_14_/CHCl_3_ volume ratio. In the ternary system of “PLAA/CHCl_3_/C_6_H_14_”, phase separation was primarily attributed to alterations in polymer concentration within both good solvent and nonsolvent at low concentrations of PLAA. This phenomenon followed the nucleation growth (NG) mechanism. However, the phase separation mechanism exhibited a greater propensity toward the spinodal decomposition (SD) mechanism at higher concentrations of PLAA [10].

## 4. Conclusions

L-arginine was utilized as a modifier and copolymerized with L-lactic acid to synthesize poly(lactic acid-*co*-arginine) (PLAA). The resulting structure and properties were then characterized. The results demonstrated the formation of amide and C–N bonds in the copolymer and confirmed the successful synthesis of PLAA, indicating that PLAA is an amphiphilic and amorphous copolymer. Ternary system solutions were prepared by selecting chloroform as a good solvent and n-hexane as a nonsolvent, and the “PLAA/CHCl_3_/C_6_H_14_” ternary phase diagram was constructed, which provided the necessary basic information for the design, processing, and application of PLAA materials with special morphologies.

## Figures and Tables

**Table 1:** Results of solution observation at different concentrations and ratios of PLAA/CHCl_3_/C_6_H_14_.

C_6_H_14_/CHCl_3_ (*v*/*v*)	PLAA in CHCl_3_ Concentration (wt%)																							
	0.25	0.5	0.75	1	2	2.25	2.5	2.75	3	4	6	7	10	12	15	15.25	15.5	15.75	16	17	19	25	30	35
0.25																					S	S	S	S
0.5														S	S	L	L	L	L	L				
0.6													S	L	L									
0.75					S	L	L	L	L	P		P		P										
1		S		S	S	P	P	P	P	P	P	P												
1.15		S		S	P																			
1.25	S	S	S	P																			P	

## Data Availability

The original contributions presented in this study are included in the article. Further inquiries can be directed to the corresponding author.
